# The role of social support and emotional exhaustion in the association between work-family conflict and anxiety symptoms among female medical staff: a moderated mediation model

**DOI:** 10.1186/s12888-020-02673-2

**Published:** 2020-05-29

**Authors:** Hui Zhang, Leiwen Tang, ZhiHong Ye, Ping Zou, Jing Shao, Man Wu, Qi Zhang, Guojin Qiao, Shaoyu Mu

**Affiliations:** 1 Zhejiang University School of Medicine Sir Run Run Shaw Hospital, Hangzhou, 310016 Zhejiang China; 2 School of Nursing, Nipissing University, Toronto, Ontario Canada; 3 Department of cardiology, Guizhou Provincial People’s Hospital, Guiyang, Guizhou China; 4 The affiliated hospital of Guizhou Medical University, Guiyang, Guizhou China; 5 Nursing College of Chongqing Medical University, Chongqing, China

**Keywords:** Anxiety symptoms, Work-family conflict, Emotional exhaustion, Social support, Female medical staff

## Abstract

**Background:**

Limited work has been done to explain how work-family conflict is related to anxiety symptoms and the roles of emotional exhaustion and social support may play.

**Methods:**

Based on a sample of 764 female nurses and physicians, a model was tested in which emotional exhaustion served as a mediator and social support was regarded as a moderator between work-family conflict and anxiety symptoms.

**Results:**

This current study supported a moderated mediation model where the relationship between work-family conflict and anxiety symptoms via emotional exhaustion was weakest for female medical staff who reported high levels of social support.

**Conclusions:**

This study contribute to providing an understanding of how and when work-family conflict affects anxiety symptoms. The results implicate a wide range of interventions aimed at promoting mental wellbeing among female medical staff for policymakers and individuals.

## Background

According to the “Healthy China 2030” program, a call-to-action highlighted for 2016–2030 is the need to “reduce mental disorders, burnout, and job-related stress, enhance stress management and introduce screening programs to promote wellbeing in the workplace” http://www.nhc.gov.cn/guihuaxxs/s3586s/201610/a2325a1198694bd6ba42d6e47567daa8.shtml. Therefore, a comprehensive understanding and effective interventions for mental health in the workplace are needed. As there has been an increase in female participation in the labor market, the World Health Organization states that more emphasis should be placed on women’s mental health concerns, such as anxiety, depression, and somatic complaints, which disproportionately affect females https://www.who.int/mental_health/prevention/genderwomen/en/. One possible explanation for this distribution of mental health concerns may be that females commonly suffer the double burden of work and family responsibilities, separation from their children, and a lack of family or co-worker support [1].

The medical profession can be one of the most stressful and demanding careers, which can lead to negative effects on mental health among physicians and nurses. A large and growing body of literature has shown that physicians and nurses have a higher prevalence of mental health disorders (eg., symptoms of depression, anxiety, and suicidal ideation) compared with the general population because of a heavy workload, the morbidity and mortality of patients, and challenging daily work routines [2]. Anxiety, which is one of the most common psychological disorders, can be more prominent among female medical professionals [3]. This mental health issue has a detrimental impact on professional performance and well-being, and may lead to issues with patient safety. A wide range of factors are likely to have an impact on the prevalence of mental disorders among medical professionals, including burnout, social support, and role stress [4].

Work-family conflict can be defined as, “An inter-role conflict in which the role pressures from the work domain are, to some extent, incompatible with the family domain” [5]. Work-family issues serve as a vital factor within the domain of occupational health psychology, especially for women [6]. Due to an increased participation of women in the labor market, the number of dual-earner families has grown substantially. However, women are frequently regarded as the primary caregivers of the family with the responsibility of housework and caring for children. Therefore, the demands of work and family make it increasingly likely for women to experience work-family conflict compared to men. Moreover, studies have shown that female medical professionals are more vulnerable to experiencing work-family conflict due to struggling with their work and family responsibilities [1, 7, 8]. Work-family conflict does not only affect women by limiting the opportunity for a leadership position in their career and their professional activity [9], but also negatively affects their mental health among female employees [10, 11]. In addition, one study demonstrated that female medical staff who suffer greater work-family conflict were more likely to develop mental health problems compared to male counterparts [12] .

Burnout is another factor that contributes to mental health issues among medical professionals [2]. Burnout refers to “a psychological syndrome emerging as a prolonged response to chronic interpersonal stressors on the job,” and it includes emotional exhaustion, depersonalization, and reduced personal accomplishment [13]. Emotional exhaustion is a feeling of excessive emotional stress and feeling drained from interaction with other people, and is the core element of burnout [14]. Higher workload is the main reason for higher emotional exhaustion [15]. As healthcare providers commonly suffer from their demanding jobs, longer work hours, and regular night shifts, they are likely to experience emotional exhaustion. Additionally, it has been postulated that emotional exhaustion is associated with work-related crisis, such as job withdrawal, dissatisfaction, and intention to leave [16]. People who suffer a high level of work-family conflict are likely to develop emotional exhaustion toward their current career [17], and emotional exhaustion is positively associated with psychological health problems [18]. Furthermore, a study has proposed that work-family conflict is significantly correlated with emotional exhaustion among female medical professionals compared with their male colleagues [19]. These findings have suggested that emotional exhaustion may have a crucial role in helping understand the relationship between work-family conflict and mental health among female medical professionals. However, much uncertainty still exists about the relationship between emotional exhaustion, work-family conflict, and anxiety symptoms among female medical professionals.

When individuals experience distress, social support can be considered to be a coping strategy [20]. Social support refers to the perceived availability of resources, involving support offered by the individual’s social networks, such as spouses, friends, co-workers, and families [21]. It has been previously shown that social support can significantly reduce the detrimental effect of stressful conditions to prevent mental problems for individuals [21]. Social support can have positive impacts on health via two effects: a direct effect and a buffering effect [21]. A large and growing body of literature has proposed that social support is negatively associated with psychological distress [22–25]. Evidence suggests that social support has beneficial impacts on anxiety symptoms among medical professionals [26]. Regarding the buffering effect of social support, previous research has established that it can be seen as a moderator between burnout and anxiety symptoms [27]. Furthermore, it has also been proposed that social support can moderate the effect of work-family conflict on burnout [28]. It is observed that the positive impact of social support is more apparent for female medical staff compared to their male counterparts [29]. There is little published data on exploring when social support moderates the relationships between work-family conflict, social support, emotional exhaustion and anxiety symptoms among female medical professionals.

This study seeks to explore the important mechanisms between work-family conflict, social support, emotional exhaustion, and anxiety symptoms among female medical professionals. The natural characteristics of gender and profession are highly correlated with work-family conflict and anxiety symptoms, and little is known about these mechanisms among this population. This study focuses on emotional exhaustion as one mechanism that may explain the association between work-family conflict and anxiety symptoms. Moreover, this study explores the moderating effect of social support in such mechanism. By doing so, potential methods to improve mental health among female medical professionals experiencing can be identified.

### Theoretical background

In line with the Job Demand-Resources Model (J-DR model), job characteristics can be classified into two categories: job demands and job resources. Job demands are ‘negative aspects’, like a heavy workload, work-family conflict, and job uncertainty [30, 31]. In contrast, job resources are defined as ‘positive aspects’ that refers to social support and room for personal growth and development [30, 31]. The J-DR model consists of a motivational process and a health impairment process [30, 31].

This study focused on the health impairment process, which implies that excessive job demands can increase the risk for burnout and result in negative outcomes, such as health problems (e.g. anxiety) [32]. Work-family conflict can be regarded as one form of job demands [33], and emotional exhaustion is the core of burnout [13]. In the health impairment process, individuals with work-family conflict find that work demands deplete their resources, which makes it difficult for them to meet familial expectations. Furthermore, inadequate recovery causes emotional exhaustion. In the long term, emotional exhaustion can drain employees’ mental and physical resources and therefore contribute to mental health problems, such as anxiety and depression [32]. This means that the effect of work-family conflict can predict emotional exhaustion, resulting in anxiety symptoms. The mediating role of emotional exhaustion in the health impairment process can be found in previous studies. For example, research conducted by Santa et al. (2018) has shown that job demands predicted higher levels of anxiety through emotional exhaustion [34]. Thus, the following hypothesize is formed:

- Hypothesis 1: The effect of work-family conflict on anxiety symptoms will be mediated by emotional exhaustion.

In the health impairment process, job resources play an important role in buffering the effect of work-family conflict on burnout [35]. Social support can be an important job resource due to its buffering effect. When stressful events have a negative effect on an individual’s wellbeing, the presence of social support can serve as a protective factor. Social support can help an individual minimize the stress they perceive or adopt healthy behaviors in response to stressors [21]. Existing research recognizes the critical role played by social support. For example, Huang et al. (2015) in a study among nurses found a moderating effect of social support on the relationship between work-family conflict and burnout [28]. Therefore, in line with the J-DR model, we consider that social support can be seen as a moderator in the health impairment process. Thus, the following hypothesize is formed:

- Hypothesis 2: Social support from private life and work life (e.g., co-workers and family) moderates the mediating effect of emotional exhaustion on the relationship between work-family conflict and anxiety symptoms.

**Fig. 1:**
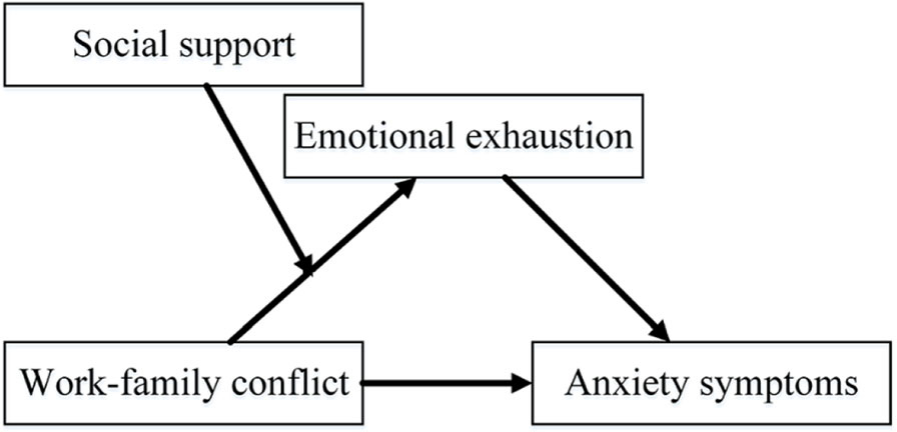
Conceptual model

Based on above, the conceptual model is presented in Fig. 1.

This research contribution is threefold. First, the issue of work-family conflict has received considerable critical attention, but far less work has been done to explain how work-family conflict is related to anxiety symptoms and the role that emotional exhaustion has plays among this population. Second, although some progress has been made in advancing the understanding of the role of social support, it is important to know if social support moderates the effect of work-family conflict on anxiety symptoms via emotional exhaustion among this population. Third, empirical evidence is provided to identify workplace interventions for policy makers.

## Methods

### Study units and participants

A convenience sample was used in this study. Data were collected by three trained researchers from May 2019 to August 2019. Female nurses and physicians were recruited from the Guizhou Provincial People’s Hospital, the affiliated hospital of Guizhou Medical University, Puan People’s Hospital, and Sir Run Run Shaw Hospital.

The researchers contacted nurse managers from the hospitals to acquire consent to participate in this study. If nurse managers agreed, they were asked to provide the number of female physicians and nurses working in their wards. A total of 861 female nurses and physicians from the hospitals were invited to participate. The researchers explained the aims of the study to each female physician or nurse, and these medical staff made an informed decision about participation in the study. Twenty participants refused participation in the study. Self-administered paper-pencil questionnaires were distributed to the remaining 841 participants. All participants were informed that questionnaires were anonymous, and the collected data were kept confidential. The survey was completed by 769 participants resulting in a response rate of 91.4%. Participants were excluded from the final sample if they failed to provide any responses to the key variables examined. The final study sample included 764 participants.

### Measures

#### Anxiety symptoms

Anxiety symptoms were measured using the Zung Self-Rating Anxiety Scale (SAS) [36]. The Chinese version of the SAS was chosen for this study [37]. The SAS has 20 items and each item is scored on a 4-point Likert scale (e.g., “I feel like I’m falling apart and going to pieces”). Higher index scores reflect higher levels of anxiety [38]. The Cronbach’s alpha was 0.71.

#### Social support

The Social Support Rating Scale (SSRS), developed by a Chinese researcher, was used to evaluate social support in this study [39]. SSRS assesses received support and perceived support from private life and work life (e.g., supervisors, co-workers, and family). There are three dimensions (objective support, subjective support, and the usage of support) in this Chinese questionnaire. Fourteen items were scored on four-point Likert scales, and these items ranged from one to four (e.g., “How many intimate friends do you have, from whom you can receive support and help?”). Higher scores reflect more social support given. The Cronbach’s alpha coefficient for SSRS was 0.70.

#### Work–family conflict

Work-family conflict was measured using the work-family conflict questionnaire [40]. The Chinese version of the work-family conflict questionnaire contains five items with a seven point Likert scale (e.g., “your job reduces the amount of time you can spend with the family”) [41]. Higher scores indicate higher levels of work-family conflict. The Cronbach’s alpha coefficient was 0.94.

#### Emotional exhaustion

The Chinese Burnout Inventory [42] was used to measure burnout. This questionnaire includes 15 items and three dimensions (emotional exhaustion, depersonalization, and reduced personal accomplishment). One of the major dimensions is emotional exhaustion. This dimension consists of five items (e.g., “I feel emotionally drained from my work”), and the subscale was adopted for this study. Higher scores indicate higher levels of emotional exhaustion. The Cronbach’s alpha coefficient was 0.94.

#### Statistical analysis

Descriptive analysis, factor analysis, and pearson correlational analyses of the four variables (anxiety symptoms, social support, work-family conflict, and emotional exhaustion) were performed using IBM® SPSS® Statistics, (Version 24, IBM Corporation, New York, NY). Model 4 and model 7 of PROCESS macro were adapted to test the significance of the mediation model (H1) and moderated mediation model (H2), respectively. The approach of PROCESS macro is based on ordinary least-squares regression [43]. The bias-corrected bootstrap procedure was applied, as this technique does not assume a normal distribution of effects, and it typically has higher power than the percentile bootstrap procedure [44]. Moreover, the bias-corrected bootstrap has a low risk of elevated Type I error rate in large sample sizes (i.e. > 500) [44]. The number of bootstrap samples was 5000 in this study. Point estimates were considered significant if the bias-corrected bootstrap 95% confidence interval did not contain zero. An “index of moderated mediation” was generated by using a bootstrap confidence interval, which was recommended by Hayes (2015) [45]. A significant moderated mediation effect means that the bias-corrected bootstrap 95% confidence interval of this index is different from zero. As the PROCESS macro also can visualize interactions, the pick-a-point approach was applied for probing interactions [46].

## Results

### Preliminary analyses

Table 1 presents the descriptive statistics, correlation matrix, and average variance extracted (AVE). Each AVE exceeded 0.50. This indicates adequate convergent validity. The square of root of AVE values (0.70–0.87) exceeded the construct correlation values (− 0.29–0.54), which suggests that discriminant validity is satisfactory.

**Table 1:** Correlation coefficient, mean, standard deviation, and AVE

Variables	M	SD	AVE	1	2	3	4
1 SS	2.68	0.78	0.50	**0.70**			
2 EE	2.75	1.51	0.75	−0.27^a^	**0.87**		
3 WFC	3.10	0.95	0.74	−0.29^a^	0.54^a^	**0.86**	
4 AS	1.68	0.53	0.62	−0.21^a^	0.34^a^	0.36^a^	**0.79**

*SS* Social support, *EE* Emotional exhaustion, *WFC* Work-family conflict, *AS* Anxiety symptoms, *AVE* average variance extracted

^a^Significant at the 0.01 level; the square of root of AVE values are bolded

### Mediation analyses

Model 4 of PROCESS macro was adapted to test if emotional exhaustion mediated the association between work-family conflict and anxiety symptoms. Results showed that work-family conflict was related to anxiety symptoms directly (b = 0.14, 95%CI [0.09, 0.18]), and work-family conflict was associated with anxiety symptoms indirectly through emotional exhaustion (b = 0.06, 95%CI [0.04, 0.09]) (Table 2). This supports hypothesis 1.

**Table 2:** Mediation analyses

DV	IV	coeff	se	t	p	LLCI	ULCI	*F*	*R*^*2*^
AS	constant	1.06	0.05	19.43	<.001	0.95	1.16	78.17***	0.16
	EE	0.08	0.01	5.27	<.001	0.05	0.10		
	WFC	0.14	0.02	6.09	<.001	0.09	0.18		
EE	constant	0.11	0.15	0.77	0.44	−0.18	0.40	307.63***	0.29
	WFC	0.85	0.05	17.54	<.001	0.76	0.95		
	Indirect effect	**0.06**	**0.01**	**–**	**–**	**0.04**	**0.09**		

Bootstrap sample size = 5000

*DV* Dependent variable, *IV* ndependent variable, *ULCI* Upper Limit of Confidence Interval, *LLCI* Lower Limit of Confidence Interval, *SS* Social support, *EE* Emotional exhaustion, *WFC* Work-family conflict, *AS* Anxiety symptoms, *Indirect effect* Work-family conflict→Emotional exhaustion→Anxiety symptoms

*** *p* < 0.001

### Moderated mediation analyses

**Fig. 2:**
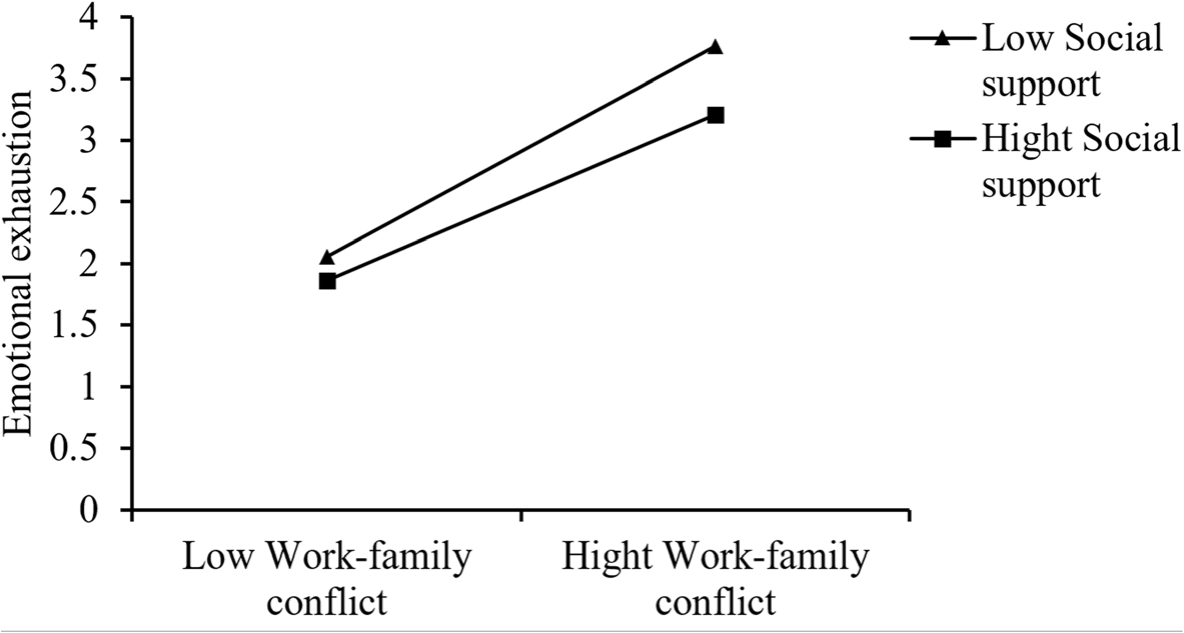
The interaction between work-family conflict and social support on emotional exhaustion

The results of model 7 of PROCESS indicated that social support moderated the relationship between work-family conflict and emotional exhaustion (b = − 0.12, *p* = 0.03). The pick-a-point measure was adopted to probe the interaction. Figure 2 further revealed that work-family conflict was more strongly related to emotional exhaustion at a low level of social support (b = 0.90, *p* < 0.001) compared to a high level of social support (b = 0.71, *p* < 0.001). As shown in Table 3, the indirect effect of work-family conflict on anxiety symptoms was strongest at the lowest (− 1 SD) level of social support and weakest for those who were at higher levels (+ 1 SD) of social support. The confidence intervals of moderated mediation index did not contain zero (b = − 0.01, 95%CI [− 0.02, − 0.01]), so this index was found to be significant. This supported hypothesis 2.

**Table 3:** Moderated mediation analyses

Values of moderators	Indirect effect	SE	LLCI	ULCI
Social support				
-1SD	0.07	0.01	0.04	0.10
M	0.06	0.01	0.04	0.09
+ 1 SD	0.05	0.01	0.03	0.08
Index of moderated mediation	−0.01	0.01	−0.02	−0.01

Outcome variable: Anxiety symptoms. Bootstrap sample size = 5000

*ULCI* Upper Limit of Confidence Interval, *LLCI* Lower Limit of Confidence Interval; Moderated mediation effect: social support moderates the indirect effect of work-family conflict on anxiety symptoms

## Discussion

Work-family conflict can be an important issue for women as females are traditionally tasked with managing familial responsibilities (e.g. child rearing, household chores), which can have a detrimental impact on their wellbeing [11]. Female medical professionals are more likely to suffer work-family conflict leading to mental problems due to an overly demanding work environment. The potential influence of work–family conflict on female medical staff has drawn increased attention from psychologists and policy makers. This study investigated how and when work-family conflict affected anxiety symptoms, and the results contribute to highlighting the need to develop programs to help female physicians and nurses. The effect of work-family conflict on anxiety symptoms through emotional exhaustion was found to be conditional on social support of this female population.

The results indicate that work-family conflict has a direct effect on anxiety symptoms. This suggests that if the stressors of work-family conflict are chronic, mental health problems may arise [47]. This is because chronic demands in one domain require sustained physical and/or mental effort. The continual depletion of energy eventually results in physiological and psychological costs, leading to negative long-term outcomes (e.g., mental health problems). These results are consistent with previous work that has observed that female nurses experiencing work-family conflict disproportionately suffer from psychophysical health conditions [48]. Therefore, considering the role of women, flexible work arrangements and part-time jobs may play a vital role in reduction of the effect of work-family conflict on anxiety symptoms among female medical staff [49].

Our results indicate that high levels of work-family conflict are indirectly associated with higher anxiety symptoms, with an increase in emotional exhaustion operating as the mediator. These findings enrich the knowledge on mediating mechanisms, explaining associations between work-family conflict and anxiety symptoms among female medical staff. A previous study also found that emotional exhaustion has significant mediating effects in the link between job stress and anxiety [50]. In line with the J-DR model, emotional exhaustion mediated the relationship between work-family conflict and anxiety symptoms. When an individual experiences work overload for long periods of time, they find they cannot fulfill their familial responsibilities. In the long term, this may drain their personal resources, including emotional and mental energy, resulting in mental health problems (e.g., anxiety) during the depletion process.

Drawing on the J-DR model, a model in which the indirect effect of work-family conflict on anxiety symptoms via emotional exhaustion is moderated by social support is proposed. The results of this study support this model and highlight the buffering role of social support in influencing anxiety symptoms through emotional exhaustion among female medical staff. Previous research also supports the moderating effects of social support on work-family conflict and burnout [28]. Moreover, Santa et al. (2018) reported that the indirect effect of job demand on anxiety can be moderated by social support [34]. Based on the J-DR model, job resources (e.g., social support) can buffer the effect of job demands (e.g., work-family conflict) on anxiety symptoms via emotional exhaustion. This is because the buffering effect of social support can change individuals’ perceptions and cognitions evoked by stressors, and moderate people’ appraisal process to stressful events and/or reduce the impact of stressors on health outcomes.

### Practical implications

Since emotional exhaustion can mediate the effect of work-family conflict on anxiety symptoms, a wide range of action for decreasing emotional exhaustion should be adopted by organizations and individuals.

From an organizational perspective, reducing job demands can be regarded as a vital step to reducing emotional exhaustion and fostering a more positive work-place environment based on J-DR theory [18]. As job demands is associated with burnout (emotional exhaustion), the creation of optimal job demands can be the key to mitigating emotional exhaustion. Hospitals should recruit sufficient human resources to enable each employee to avoid suffering an overly demanding workload and exceedingly high levels of work pressure. Therefore, a supportive organizational climate could help promote well-being while simultaneously reducing emotional exhaustion. From an individual’s perspective, certain personality traits can be beneficial, such as emotional stability, conscientiousness, and agreeableness, which can help employees perceive their work environments favorable despite of the challenging nature of their jobs [18]. Related training modules can be designed to teach employees how to improve individuals’ personality. Additionally, other individual-level interventions are effective, such as job crafting training, strengths use training, and recovery training [18]. Individuals can adopt these measures to learn how to proactively change their work environment to avoid experiencing emotional exhaustion by setting personal goals, using their strengths for their career, and using relaxation or mindfulness techniques [2].

This study indicates that social support can be a useful resource for female healthcare providers to improve mental health. Individuals can obtain support from their social network, such as coworkers, supervisors, and family. Based on the traditional Chinese culture, it is widely believed in Chinese society that women should mainly contribute their time and efforts to the familial domain, regardless of their career development. However, female medical professionals could achieve positive work-life balance if other family members provided a larger proportion of contributions [51]. In particular, grandparent-provided childcare can also be an option, as well as high-quality formal childcare services in Chinese dual-career couples, and women’s partners can also take more responsibilities for housework in their spare time [52].

Furthermore, supervisors and colleagues from a supporting network are crucial to female medical staff. Supportive supervisors can help female nurses and physicians handle critical patients properly and effectively by providing personal coaching. These supervisors can also provide them with the confidence to achieve high-quality standards in their work across many different tasks [53, 54]. Meanwhile, supportive colleagues can offer help to cover additional work when female nurses and physicians suffer from irregular and long working hours. The understanding and support from colleagues can enable female nurses and physicians to relax and feel less anxious, so they are less likely to develop emotional exhaustion and anxiety.

### Study limitations

It is important to consider some limitations to this study. First, this was a cross-sectional study, so causal relationships between these variables cannot be established and the reciprocal direction of the relationships cannot be determined based on the results. For example, anxiety symptoms could also influence burnout. Longitudinal and experimental research in the future are required to draw reliable and firm conclusions. Second, self-reported measures were adopted in this study, which may raise concerns about common method variance causing an overestimation. Future studies are encouraged to adopt objective rather than subjective measures. For example, criteria of Diagnostic and Statistical Manual of Mental Disorders (DSM) can be used by mental health professionals to diagnose anxiety. Third, the direction of family-to-work conflict was not considered, which may also play a vital role in the process of the effect of work-family conflict on anxiety symptoms. However, this study focused on the direction of work-to-family conflict, because this direction is more likely to relate to job demand [55], which is one of the core factors of emotional exhaustion. Additionally, the moderating effect of social support in this study was tested, but other personal resources (e.g., self-efficacy and optimism) that may be effective should also be considered in the future based the proposed model. Fourth, future studies should examine whether work-family conflict could predict anxiety symptoms via other dimensions of burnout (e.g., depersonalization and reduced personal accomplishment). Fifth, control variables were not included in this study. However, a critical review suggested that researchers should include control variables based on theoretical justifications, rather than early empirical associations or defaulting to “they might associate” with the investigated variables [56]. Finally, the results are only based on females, which means that the findings cannot be generalized to both genders in hospitals. Future studies could incorporate females and males into this proposed model to explore the relationships between these key factors.

## Conclusion

These findings suggest a role for emotional exhaustion and social support in promoting mental health among female medical staff. Policymakers should realize that healthy female employees can perform better at hospitals, and thus consistently contribute towards accomplishing organizational goals. Necessary measures should be adopted to effectively support them. Female medical staff need to raise awareness of the role of social support and emotional exhaustion, which can affect their mental health.

## Data Availability

The datasets used and analyzed during the current study are available from the corresponding author on reasonable request.

## Abbreviations

CI: Confidence Interval
OR: Odds Ratio
DV: Dependent variable
IV: Independent variable
ULCI: Upper Limit of Confidence Interval
LLCI: Lower Limit of Confidence Interval
SPSS: Statistical Package for Social Sciences
SSRS: Social Support Rating Scale
SAS: Zung Self-Rating Anxiety Scale
SS: Social support
EE: Emotional exhaustion
WFC: Work-family conflict
AS: Anxiety symptoms
